# Application of Amino Acids for High-Dosage Measurements with Electron Paramagnetic Resonance Spectroscopy

**DOI:** 10.3390/molecules28041745

**Published:** 2023-02-12

**Authors:** Yordanka Karakirova

**Affiliations:** Institute of Catalysis, Bulgarian Academy of Sciences, 1113 Sofia, Bulgaria; daniepr@ic.bas.bg

**Keywords:** electron paramagnetic resonance (EPR) spectroscopy, amino acids, proline, cysteine, alanine, dosimetry, free radicals, γ-radiation

## Abstract

A comparative investigation of amino acids (proline, cysteine, and alanine) as dosimetric materials using electron paramagnetic resonance (EPR) spectroscopy in the absorbed dosage range of 1–25 kGy is presented. There were no signals in the EPR spectra of the samples before irradiation. After irradiation, the complex spectra were recorded. These results showed that the investigated amino acids were sensitive to radiation. In the EPR spectrum of cysteine after irradiation, RS• radicals dominated. The effects of the microwave power on the saturation of the EPR signals showed the presence of at least three different types of free radicals in proline. It was also found out that the DL-proline and cysteine had stable free radicals after irradiation and represented a linear dosage response up to 10 kGy. On the other hand, the amino acid alanine has been accepted by the International Atomic Energy Agency as a transfer standard dosimetry system. In view of this, the obtained results of the proline and cysteine studies have been compared with those of the alanine studies. The results showed that the amino acids proline and cysteine could be used as alternative dosimetric materials in lieu of alanine in a dosage range of 1–10 kGy of an absorbed dose of γ-rays using EPR spectroscopy. Regarding the radiation sensitivity, the following order of decreased dosage responses was determined: alanine > DL-proline > cysteine > L-proline.

## 1. Introduction

Among the various methods of dosimetry, for example, polarimetry, photo- and thermo-luminescence, measurements of electroconductivity and dielectric losses, etc., EPR dosimetry has particular significance. In a number of cases, EPR spectroscopy has shown advantages over the other methods. These advantages include a high sensitivity for a wide measurement range with high accuracy, the small size of the used samples, the non-destructive character of the measurements, and the automation of the processing of the dosimetric data. Many scientists have made valuable contributions to the development of reference standard dosimeters for high energy radiation on the basis of using alanine in an EPR dosimetry system [1,2]. Until the present, this has been the most common material used in EPR dosimetry, and it has been formally accepted by International Atomic Energy Agency (IAEA, Vienna, Austria) [3], the National Institute for Standards and Technology (NIST, Maryland, USA) [4], and the National Physical Laboratory (NPL, Teddington, UK) [5] as a secondary reference and transfer dosimeter for high-dosage irradiation. Alanine EPR dosimetry has been applied successfully for measuring intermediate and high radiation doses. Although the performance of alanine dosimetry has improved, the sensitivity of the material is too low for a fast and simple low-dosage determination. However, there are widely spread applications of alanine, and many scientists continue to search for alternative materials with better characteristics. Materials with greater sensitivity are required to make EPR dosimeters competitive with other dosimetry systems. Strategies for identifying new EPR dosimeter materials have been proposed by Ikeya et al. and Lund et al. [6,7]. The criteria that should be fulfilled by a useful dosimetry system can be divided into radiation dosimetry criteria and radiation chemistry criteria in regard to EPR properties. The important radiation dosimetry criteria are tissue equivalence (with respect to scattering) and the energy absorption of ionizing radiation, as well as the stability of radicals over time and the linearity of the signal versus the dose. The radical stability and linearity of the signal with respect to dose must be verified experimentally, and tissue equivalence excludes materials containing heavy elements. The important radiation chemistry criteria are a high radical yield and a suitable radical structure, which provide longer-living radicals with a simple symmetrical line and a short longitudinal relaxation time period. Many studies have already been carried out in an effort to identify new materials for electron paramagnetic resonance dosimetry and to substitute alanine in such dosimetry [7,8,9,10,11,12,13,14,15,16]. Many substances, such as saccharides [17], formates [18,19], tartrate [20], dithionate [21], and ascorbic acid [22], have been studied as dosimetric materials, and a number of amino acids in which free-radical populations form during irradiation have been suggested for high-dosage dosimetry using electron paramagnetic resonance analysis. Several compounds, all of which have been found to be more sensitive than alanine by a factor of 2–10, have been investigated [23,24]. Sucrose has also been widely studied as a dosimeter in radiation accidents, for irradiation with different types ionizing radiation, and with mixed types of radiations [17,25,26,27,28,29,30]. In the current study, the dosimetric properties of the amino acids proline and cysteine were investigated and compared with those of alanine. These materials were chosen because they are known to have good characteristics as dosimetric materials. They belong to a class of biological substances which, after irradiation, exhibit reasonably well-resolved spectra. It is convenient to study them by EPR spectrometry for two reasons: amino acids are components of proteins that can be purified in crystalline form, and they are used in many foods and food additives. Because of this, if they show good results, they could potentially be used for accidental and/or retrospective dosimetry. The application of the amino acids to a successful and versatile free radical method of dosimetry depends on the magnitude of the radical yield per unit of absorbed dose and on the lifetime of the free radicals. On the other hand, proline and cysteine are among the few left amino acids that have not yet been studied with respect their use in EPR dosimetry. In view of this, in the present study, all characteristics for dosimetric materials such as sensitivity to radiation, time stability of the radiation, the created free radicals, and the dose–response characteristics of proline and cysteine were studied. The obtained results showed the possibilities of using these materials for dosimetric purposes for γ radiation, and this study will enrich the existing knowledge about the EPR dosimetry of amino acids.

## 2. Results and Discussion

### 2.1. EPR Spectra

**Figure 1:**
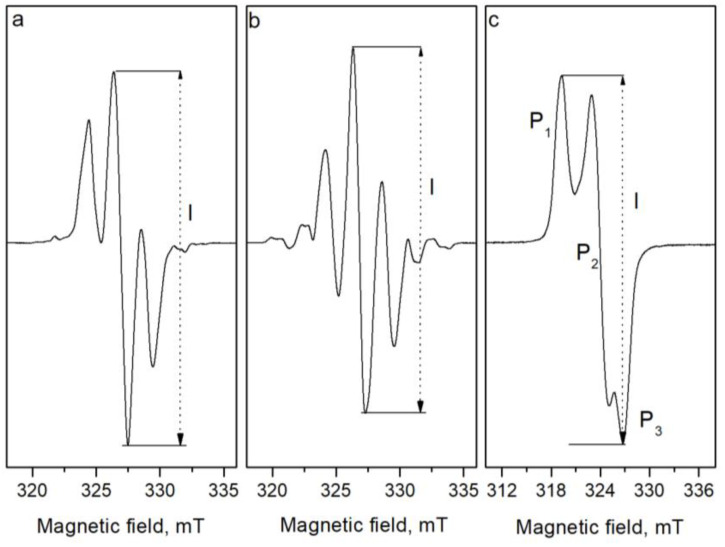
EPR spectra of (**a**) L-proline, (**b**) DL-proline, and (**c**) cysteine after *γ*-irradiation with 10 kGy.

No EPR signals were observed in the samples before irradiation. After irradiation, complex EPR spectra were recorded. It is known that complex spectra are composed as a result of the superposition of the signals of several free radicals. The spectra of L- and DL- proline are shown in Figure 1a,b. As can be seen, the EPR spectra of L- and DL-proline are similar but not exactly the same. The EPR spectrum of DL-proline is characterized by a g factor of 2.00378 *±* 0.00002 of the central line, a constant of the hyperfine splitting of A ≈ 2.171, and a linewidth of ΔH ≈ 0.94 mT. The EPR spectrum of L-proline is also centered at a g value of 2.00379 *±* 0.00002, and its most intensive three lines have widths of ΔH ≈ 0.96 mT and a splitting value of A = 2.107. As DL-proline is a racemic mixture of the isomers D- and L-proline, it was not expected to have a different EPR spectrum than that of L-proline. The difference was explained based on the type of sample. In comparison with the DL-proline sample, which was crystalline, the L-proline sample was in the form of powder. This supposes a higher hygroscopicity of the sample. It is known that the absorbance of moisture from the air leads to decreases in the quantity of free radicals and the intensity of the EPR signal, respectively, because of recombination processes. Likely, some of the radicals were more sensitive to the moisture and they disappeared because of the recombination, and this change the shape of the spectra at all.

**Figure 2:**
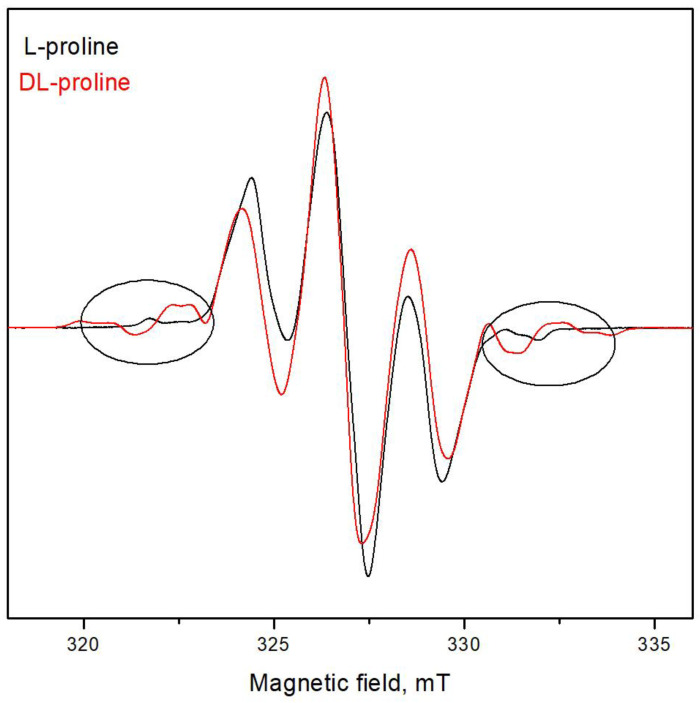
EPR spectra of L- and DL-proline irradiated with 25 kGy.

**Figure 3:**
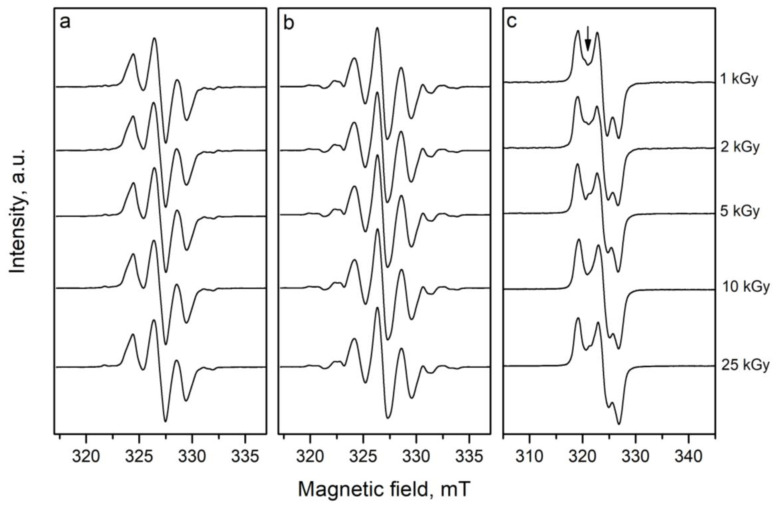
Effect of the dose on the shape of the EPR spectra: (**a**) L-proline, (**b**) DL-proline, and (**c**) cysteine.

To compare both spectra, Figure 2 shows the EPR spectra of DL- and L-proline irradiated with 25 kGy. It can be seen that some additional lines are observed at the low and high magnetic fields in the EPR spectra of DL-proline. Besides these extra lines, a small difference in the linewidths of the two lines on the left and on the right of the central line is observed. This can be related to the various relaxation times that characterized the interaction of the electron spin with the surroundings and with each other. Thus, in some cases, the lifetime of the individual spin-orientation state in the radical, or that of the radical itself, may be so short time that the linewidth is affected. These effects can arise from the electron exchange and transfer between molecular species. However, this is not so important for the aim of the current study because there were no observed differences between the spectra of the samples of the L-proline irradiated with different doses and the spectra of the DL-proline samples irradiated with different doses. Figure 3 shows the spectra of the investigated samples irradiated with different doses of gamma rays. The fact that there are differences between the spectra of the various materials did not influence the results with respect to their dosimetric properties because they are being studied as independent dosimetric materials. Since there were no changes in the EPR spectrum with the dosage, an amplitude of the first derivative (“peak-to-peak”, from maximum to minimum, denoted by I in Figure 1) can be taken as a relative measure of the quantity of the free radicals. The EPR spectra of cysteine consisted of three lines with the g factors g_1_ = 2.0542, g_2_ = 2.0251, and g_3_ = 2.0053, which are denoted by P1, P2, and P3, respectively, in Figure 1c. According to the literature data, the spectrum of cysteine after irradiation is due to the domination of RS• radicals [31]. This “sulphur pattern” is also found in the spectra of various thiols and in compounds containing S-S bonds after gammairradiation at room temperature. An interaction with one or two methylene protons in RCH_2_S∙ radicals may be observed, though, generally, the proton interactions are too small to further characterize the trapped species. However, a low intensive signal located between P1 and P2 in the spectrum, due to another type of radical with an unknown nature, which was more visible and is denoted by the arrow in Figure 3, was also observed.

The well-known powder EPR spectrum of irradiated *α*-alanine consists of five broad lines having intensity ratios of 1:4:6:4:1, separated by approximately 2.5 mT (though this is not shown). The observed quintet spectrum is attributed to the hyperfine interaction of the unpaired electron with four protons—three protons from the methyl group and one proton from the *α*-carbon atom—CH_3_C∙HCOOH. The peak-to-peak amplitude of the most intensive central line is commonly used to monitor the dosage deposited in alanine samples after exposure to ionizing radiation. Many studies on the composition of the alanine EPR spectrum are available in the literature [32,33,34,35]. It has been shown that the EPR spectrum of irradiated alanine consists of at least three different radical species [36,37,38].

### 2.2. Effect of the Dose on the Shape of the EPR Spectra

As mentioned above, Figure 3 shows the effect of the dosage on the shape of the signals in the EPR spectra. It is shown that in the spectra of L- and DL- proline, the different doses do not lead to any changes in the shape of the spectra. At the same time, a small change in the intensity of the central line in the spectrum of cysteine, relative to both lines from right and left, with the increased doses was observed. This can likely be produced by different densities of the radiation beams during irradiation with lower (1 kGy) and higher (25 kGy) doses. However, this may also be due to saturation at high dosages of the radicals responsible for this line. Previous investigations have shown that different dosages do not impact the shape of the spectra of alanine.

### 2.3. Effect of the Microwave Power on the Shape and on the Saturation Degree of the EPR Signals

**Figure 4:**
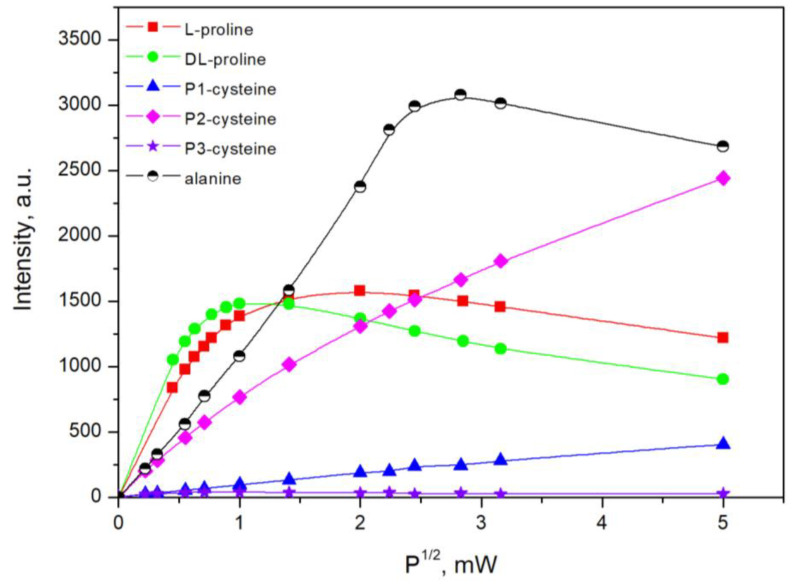
The dependence of the EPR intensity on the square root of the microwave power.

**Figure 5:**
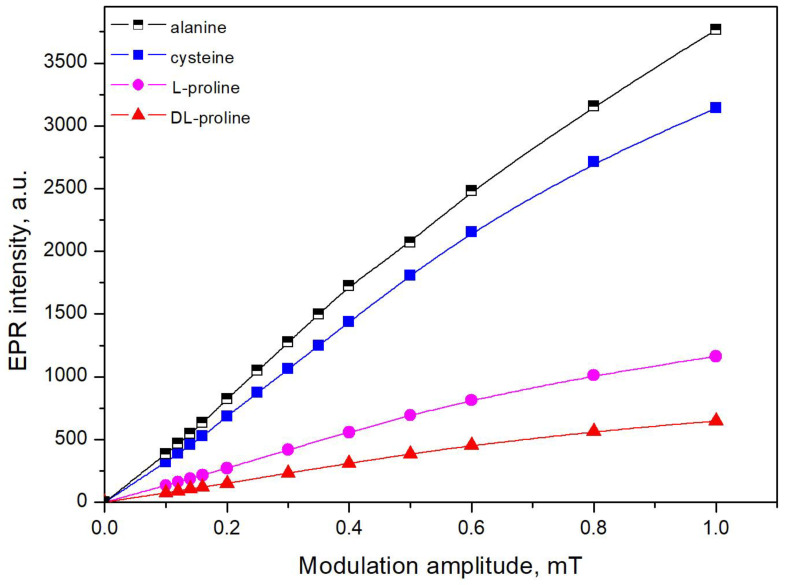
The dependence of the EPR signal intensity on the modulation amplitude.

The intensities of the EPR signals are known to depend on the values of the instrumental settings, i.e., the microwave power and modulation amplitude. Therefore, the first step after irradiation was to study the influence of these parameters on the EPR response. In view of this fact, two series of investigations on the dependence of EPR intensity as a function of the square root of the microwave power and of the magnetic field modulation amplitude were made. The results (Figure 4 and Figure 5) showed that for proline, the EPR intensity remained linearly dependent on the microwave power up to 0.3 mW and on the modulation amplitude up to 0.4 mT. For cysteine, the sample peaks 1 and 3 had linear dependence up to 6 mW, whereas peak 2 was saturated at a lower value of the microwave power (1 mW). The dependencies on the modulation amplitude were linear up to 0.4 mT. The values of the parameters that were chosen for the measurements were required be in the linear parts of the graphs. However, the appropriate instrumental settings to record the EPR spectra of alanine were previously studied to compare the spectra, and it was acceptable to determine them using the same instrument. Therefore, the following values of the parameters were identified: in the case of proline, a microwave power 0.3 mW and a modulation amplitude 0.4 mT were applied, and for cysteine and alanine, microwave power of 1 mW and modulation amplitude of 0.4 mT, respectively, were applied.

**Figure 6:**
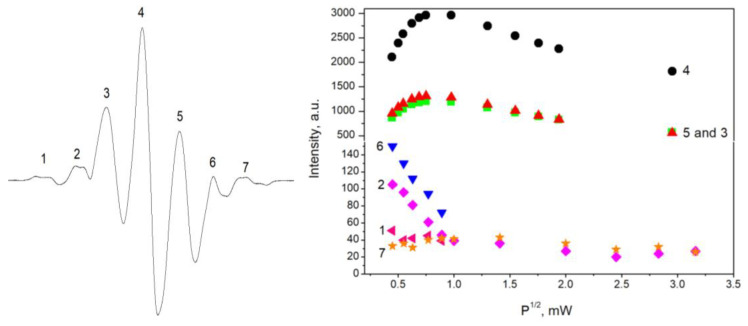
Saturation behaviors of the EPR lines in the spectra of proline versus the square root of the microwave power.

Figure 6 shows the behavior of the saturation of the EPR signal of the observed lines in the spectra of proline with regard to the microwave power. For this study, a sample of DL-proline irradiated with 5 kGy was used. The results for L-proline were the same, which is why they are not shown. The number of each line can be seen on the left panel in the figure, whereas on the right, the dependence on the square root of the microwave power is shown. As seen in the figure, peaks 3, 4, and 5 have similar behaviors, namely, a linear dependence up to 0.3 mW, which slowly decreased after that point. Peaks 2 and 6 decreased with the increase in microwave power. When the power exceeded 1 mW, peak 6 disappeared. The changes in the magnitude of the microwave power weakly influenced the intensity of peaks 1 and 7. At values higher than 1 mW, peak 1 was not observed. On the basis of these results, it can be concluded that at least three types of free radicals were created in proline during the γ-irradiation. The first one was responsible for peaks 3, 4, and 5 in the EPR spectra of proline. Peaks 2 and 6 in the spectra are due to the second type of radical. The last radical was responsible for peaks 1 and 7. Similar to this, if we look at the dependence of different peaks in the spectra of cysteine with regard to the microwave power (Figure 4, P1, P2, and P3), it can be seen that two different saturation behaviors were observed. One of them was for peaks P1 and P3 and the second was for P2. Therefore, this is evidence that in addition to RS• radicals in the EPR spectra, there are also contributions by other paramagnetic species with unknown nature. This statement is in accordance with the observation in Figure 3, where it can be seen that P2 had changed its intensity regarding P1 and P3 after the increase in the dose.

The investigation of the saturation effect of the lines in the EPR spectra of alanine upon increase in the microwave power showed the presence of three different types of radicals (R1, R2, and R3) [36].

### 2.4. Time Dependence of the Free Radicals

The time stability of free radicals depends on their molecular surroundings and, especially, on the state of the atom. Normally, in solutions, the lifetimes of the unpaired electrons or free radicals are very short. Some crystalline materials, even at room temperature, can exist for as long as several years. The type and amount of the free radicals created by ionizing radiation depend on the crystalline structure and storage conditions.

From a dosimetry point of view, the knowledge of the time stability of the radiation-induced EPR signal in the samples, as well as their decay kinetics, are highly important. This is especially important when several days can elapse between the exposure and the instrumental evaluations. There are at least two mechanisms of decay in the monitored free radicals: recombination with other paramagnetic species to create diamagnetic products and their transformation into another paramagnetic molecule. In the first case, only the intensity of the obtained EPR spectrum will decrease with time and no changes in its shape will be observed, and in the second case, new EPR spectra will appear. As it is typical, the effects of the second type were observed immediately after irradiation of the substance under study until stable paramagnetic species were formed. After that, the remaining stable free radicals could only recombine. In view of this, all measurements were performed at least 72 h after irradiation in order to avoid short-living intermediate relaxing products.

**Figure 7:**
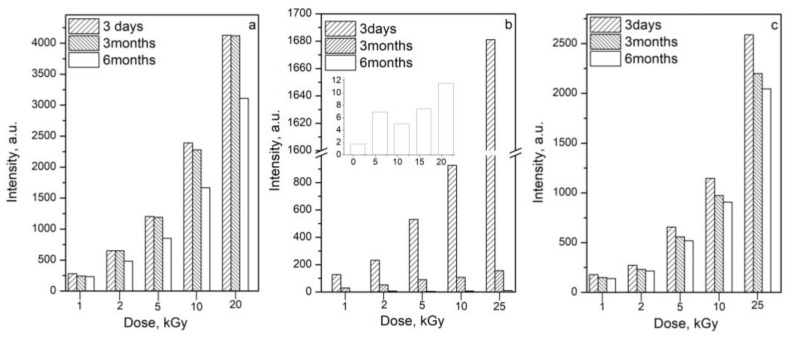
Time stability of the free radicals recorded for: (**a**) DL-proline; (**b**) L-proline, where the small graph shows the EPR signal intensity after six months of storage; and (**c**) cysteine.

For this study, the samples were stored at room temperature in the dark and then measured for a period of six months. The results showed that for this period, the intensity of the DL-proline decreased by approximately 25% (Figure 7a). The radiation-induced signal of L-proline decreased by 83% for 3 months (Figure 7b). Six months after irradiation, the signals had decreased by 99% and nearly disappeared. This result can be explained by the fact that the samples of L-proline are more hygroscopic than DL-proline. The L-proline was in the form of powder, whereas the DL-proline samples were crystals. However, the samples were stored under the same conditions, it was visible that the samples of L-proline had absorbed moisture from the air, even though they were stored in plastic bags in a dry and dark place. In the results, the recombination of the free radicals was observed, and therefore, there was a decrease in the signal intensity.

The cysteine intensity decreased by 21% over the same time period of six months (Figure 7c).

The investigations of the lifetimes of the radiation-induced radicals in alanine were completed more than 35 years ago. They showed that the lifetimes of the free radicals were very long [39]. It was reported that the decay rate was less than 1% for 3 years. On the other hand, Hansen and Olsen [40] found a dependence between radical decay and applied dosage. They discovered very low fading for doses below 10 kGy and more pronounced fading for doses above 50 kGy. This showed that the radicals could likely begin to interact with each other above a certain concentration. This fact can also explain the saturation of the signal intensity at higher doses. There have been many other studies on the time stability of irradiation-induced free radicals in alanine, and as a whole, they concluded that they were stable for a long time period.

### 2.5. Dose–Response Characteristics

**Figure 8:**
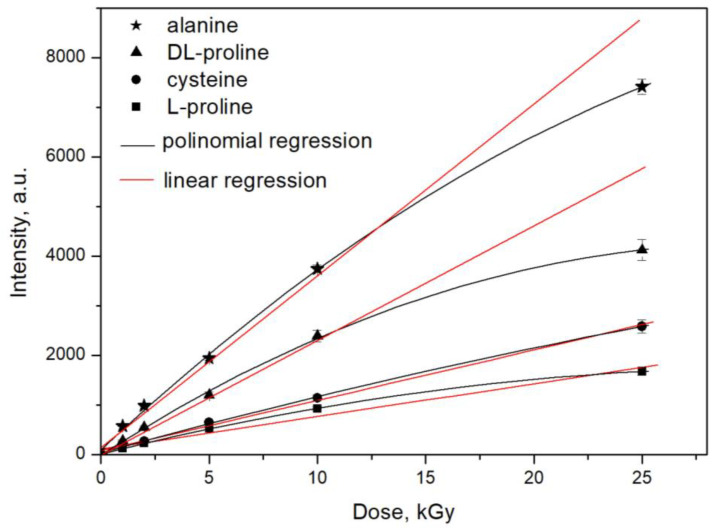
Dependence of the EPR signal intensity on the applied dose of gamma radiation.

The dose–response characteristics of proline, cysteine, and alanine were obtained for ^137^Cs γ-rays. The responses were expressed as changes in the EPR signal intensity (“peak-to-peak” amplitude of the first derivative, denoted by “I” in Figure 1) of the irradiated samples as a function of the absorbed dose. The dose –response curves are shown in Figure 8. Each data point consists of three independent measurements of three separate samples that were simultaneously irradiated.

The results showed the linear dependence of the EPR signal intensity on the absorbed dose gamma rays up to 10 kGy and the saturation of the intensity at 25 kGy. These results are in accordance with those published in the literature data for the dose–response characteristics of other materials, for example, those of mono- and di-saccharides [6]. With respect to radiation sensitivity, the following order of decreases in sensitivity was determined: alanine > DL-proline > cysteine > L-proline. The dose–response curves were built with the data obtained 72 h after irradiation. This was necessary to avoid the short-living intermediate relaxing products in the first hours after radiation treatment.

Because of the saturation processes, two types of equations were used to fit the results: linear and polynomial regression. The obtained data are shown in Table 1.

## 3. Materials and Methods

### 3.1. Materials

**Scheme 1:**
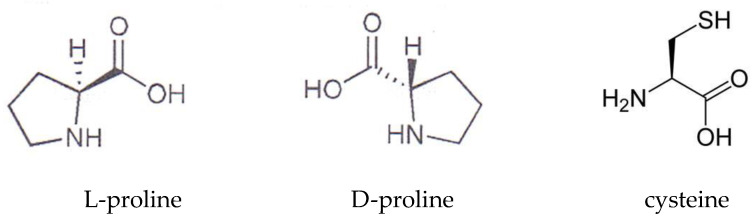
Chemical structures of proline and cysteine.

The amino acids (L-proline, DL-proline, alanine, and cysteine) were bought from Sigma Aldrich. Both forms of proline—L- and DL-—were used. The chemical structure of cysteine and the isomers of proline are shown in Scheme 1. The structure of alanine is not shown because it is well known.

L-proline is the only proteinogenic amino acid that is a secondary amine, meaning that its amine nitrogen is bound to two alkyl groups. It is especially important in the production of collagen, which is a primary component in skin, cartilage, and bones. L-proline can be found in a large number of food supplements to support the growth of connective tissue. DL-proline is a racemic mixture of the naturally occurring isomers of L and D-proline. D-amino acids have been found in relatively high abundance in human plasma and saliva.

Cysteine is a sulfur-containing, semi-essential proteinogenic amino acid. It can be synthesized by the human body under normal physiological conditions if a sufficient quantity of methionine is available. The majority of L-cysteine is obtained industrially through the hydrolysis of animal materials, such as poultry feathers or hog hair. Cysteine, primarily the L-enantiomer, is a precursor in the food, pharmaceutical, and personal care industries. One of its largest applications is in the production of flavors.

Alanine is the simplest α-amino acid after glycine. The methyl side-chain of alanine is non-reactive, and therefore, it is rarely directly involved in protein function. Alanine is a nonessential amino acid, meaning it can be manufactured by the human body, and it does not need to be obtained through the diet. Alanine is found in a wide variety of foods, but it is particularly concentrated in meats.

### 3.2. Irradiation

Three parallel samples of each amino acid were taken and irradiated with gamma rays. The irradiation was performed by applying a source, ^137^Cs, a dosage rate of 200 Gy/h, and a dosage range of 1–25 kGy. For control of the absorbed dose distribution, we used the alanine dosimeters of a Kodak BioMax. Three dosimeters were placed at each point. The control measurements and calibration of the absorbed dosage in water were completed by an X-band EPR spectrometer (E-scan, Bruker). The irradiation was performed in air and at room temperature. After irradiation, all the samples were kept in closed plastic bags at room temperature and stored in the dark.

### 3.3. Principles of the EPR Method

EPR spectroscopy can be defined as the resonant absorption of electromagnetic energy in paramagnetic substances by the transition of the spin of an unpaired electron between different energy levels (a state of lower energy and a higher-energy state), in the presence of a magnetic field. In the presence of an external magnetic field, the spin of unpaired electron is orientated to it in two directions: parallel and antiparallel to the field. The energy difference, *ΔE,* between these levels is proportional to the Lande *g*-factor, the Bohr magneton, *β,* and the magnetic field, *H*. The relationship is given by the equation:

*ΔE* = *gβH*

In case of thermal equilibrium, the population of the lower energy level E_1_, is slightly higher than that of the upper level, E_2_. Therefore, the system is able to absorb energy, *ΔE = hν,* from an external high-frequency field. When the sample is irradiated using radiation with an appropriate frequency and *hν = gβH*, transitions from the lower to the upper state appear and the EPR spectrum, as a first derivative of the absorption curve, is recorded.

### 3.4. Instrument

The EPR spectra were recorded using a JEOL JES FA 100 EPR spectrometer at room temperature. The X-band EPR spectrometer was operated at 9.5 GHz of frequency, and it had a standard TE_011_ cylindrical resonator.

### 3.5. Procedure of Measurement

For each single measurement, an equal weight of the samples was placed in quartz tubes (4 mm inner diameters). For the best sensitivity, the tubes were positioned in the center of the EPR cavity. Three independent measurements were used for every sample, including a procedure where inserting-removing-inserting of the sample was performed in the cavity of the EPR spectrometer. The data were averaged, and in that way, the error of the measurement was determined to be 3%. A reference sample, Mn magnetic diluted in MgO, which is an internal standard in the above-mentioned spectrometer, was analyzed before and after each series of measurements under the same conditions as those used for the sample measurements to normalize the signal intensity of the samples and to minimize the error resulting from any instability in the spectrometer. The parameters for recording the spectra were as follows: modulation frequency of 100 kHz, microwave powers of 0.3 mW (proline) and 1 mW (cysteine and alanine), modulation amplitude of 0.4 mT, time constant of 0.03 s, and sweeping time of 2 min.

## 4. Conclusions

After irradiating DL-proline, L-proline, and cysteine samples with γ-rays, complex EPR spectra of all samples were recorded. The effect of the microwave power on the shape and the saturation of the EPR signals showed that at least three types of free radicals with unknown natures were created in proline during the γ-irradiation, and two radicals were created in cysteine, one of which was RS•. The time dependence analysis of the EPR spectra after irradiation shows a fading of intensity of DL-proline with 25 %, L-proline – 99% and cysteine – 21% for six months. For comparison, the free radicals created by the radiation in alanine were stable for a longer time period. For all materials, the EPR signal amplitude had a linear dose response up to 10 kGy, and it was saturated at higher doses. Under the same experimental conditions, alanine also showed a linear response up to 10 kGy but with better sensitivity. All these results provide an opportunity for DL-proline and cysteine to be used as dosimetric materials for doses ranging from 1 to 10 kGy, but they have lower sensitivity than alanine. However, in case of emergency dosimetry, if they are present in such a situation, they could be successfully used for dose assessment. For retrospective dosimetry, they are not very suitable because of the decay rates of the radiation-induced free radicals. On the basis of the conducted research, it can be concluded that alanine remains the best candidate for a universal dosimetric material.

## Figures and Tables

**Table 1:** Coefficient values for the dependencies of the EPR signal intensity on the applied dosage.

Sample	Linear Regression I = A + BD			Polynomial Regression I = A + B_1_ + B_2_D^2^			
	A	B	R	A	B1	B2	R
DL-proline	251.45	163.81	0.9872	17.93	279.50	−4.60	0.9984
L-proline	110.58	65.94	0.9865	14.32	109.46	−1.71	0.9997
Cysteine	77.41	101.76	0.9984	33.32	121.70	−0.78	0.9992
Alanine	360.99	290.95	0.9944	97.55	410.05	−4.69	0.9991

I—intensity of the EPR signal; A, B—coefficients; D—the absorbed dose of radiation in kGy; R—correlation coefficient.

## Data Availability

The data analyzed in this study are available from the corresponding author upon reasonable request.

## Sample Availability

Not available.
